# A Study Protocol to Explore and Improve Access to Medical Services and Information for Recently Diagnosed Elderly Patients with Cancer in Rural Settings

**DOI:** 10.29337/ijsp.144

**Published:** 2021-04-30

**Authors:** Gianina-Ioana Postavaru, Bethan Myers, Tanweer Ahmed, Douglas Lewins, Rosemary Brown, Helen Swaby

**Affiliations:** 1 Bishop Grosseteste University, Lincoln, UK; 2 United Lincolnshire Hospitals NHS Trust, Lincoln, UK; 3 Leicestershire Partnership NHS Trust, Leicester, UK; 4 Health Research Institute, Sheffield Hallam University, Sheffield, UK; 5 National Institute for Health Research, UK

**Keywords:** rural health, oncology, pandemic, patients, healthcare professionals

## Abstract

**Introduction:**

This two-phase study seeks to contribute to research in the field of rural cancer health; specifically, the aim is to gain insight into the experiences of seeking, accessing and using information and health services throughout the cancer journey (diagnosis, treatment and follow-up care) for recently diagnosed (≤6 months) older patients (≥65 years) in rural areas.

**Methods and analysis:**

Data will be collected through in-depth semi-structured interviews. In phase 1 (before 23^rd^ March 2020) interviews were conducted with healthcare professionals (HCP) to explore their experiences of delivering care to their elderly patients. In the second phase (starting January 2021) we will conduct interviews with cancer patients to understand the impact of COVID-19 and shielding on their experiences of being diagnosed, attending appointments and accessing and receiving support from community organisations and informal support from family and friends. Data gathered will be analysed using the Framework Method.

**Ethics:**

The study has been approved by the Health Research Authority and the United Lincolnshire Hospitals NHS Trust. Initial favourable ethical opinion was granted on 1^st^ October 2019. Second favourable ethical opinion for amendments to reflect the impact of COVID-19 was received on 10^th^ August 2020. The study protocol has been registered on Research Registry.

## 1. Methods and Analysis

### 1.1. Study setting and participants

Healthcare professionals and recently diagnosed cancer patients will be recruited in Lincolnshire through medical and local patient organisations.

### 1.2. Sampling method

A purposive and snowballing sampling strategy is used based upon recommendations in the literature [1]. Ongoing debate exists around sample sizes and data saturation in qualitative research, with some authors recommending a sample size of at least 12 [2]. Little that is ‘new’ arises out of transcripts of interviews with 20 or so people [3]. The principle of saturation [4] will be used in that the data collection will stop when new data does not shed any further light on the research topic [5,6].

Principal exclusion criteria for cancer patients are: 1. Advanced cancer; 2. Age <65 years when diagnosed and receiving treatment; 3. Diagnosis under investigation but not yet confirmed by HCP; 4. >6 months since diagnosis; 5. Urban living <10 miles from treatment centre; 6. Lacking capacity to consent for themselves due to illnesses involving severe cognitive impairment (e.g. dementia, Alzheimer, Parkinson etc); 7. Not able to understand and communicate in English.

### 1.3 Procedure

***Table 1*** below highlights the steps followed by participants as part of the research protocol.

**Table 1:** Recruitment and data collection procedures.


PROCEDURE		TOTAL NUMBER OF PROCEDURES PER PARTICIPANT	AVERAGE TIME PER PROCEDURE

Healthcare professional	Telephone call to prospective HCP participant to discuss the study, eligibility criteria, answer questions, and arrange an interview	1	15 minutes

	Participants will be explained in person the Patient Information Sheet and Consent Form. Interview consent form completed in printed forms by potential participants and returned to researcher	1	30 minutes

	Face to face, in-depth and semi-structured interviews with HCP participant (includes debriefing)	1	60 minutes

Patient	Patient participants’ pre-screening: in person by clinical teams or via email/telephone by the researcher	1	5 minutes

	Telephone call to prospective patient participant to discuss the study, eligibility criteria, answer questions, and arrange an interview	1	15 minutes

	Participants will be sent an electronic study information pack explaining the Patient Information Sheet and Consent Form. Verbal consent will be obtained and recorded with audio recording equipment prior to data collection	1	30 minutes

	In-depth and semi-structured interviews with patient participant over telephone, WhatsApp or Skype	1	60 minutes


### 1.4 Data collection method

#### 1.4.1 Data collected in phase 1

- Face to face in-depth and semi-structured interviews have been conducted with 17 healthcare professionals and their colleagues (including oncologists/haematologists and Macmillan nurse specialists, medical secretaries, Cancer services attending Multidisciplinary Team Meetings) to explore their experiences of providing information and advice to this category of patients.

### 1.4.2 Data to be collected in phase 2

- Phone, WhatsApp or Skype interviews with approximately 20 cancer patients to understand their experiences of accessing and receiving effective and timely information from health providers within the past 6 months.
- Clinical care teams and patient organisations’ peer leaders will be made aware of the study and will make the initial approach to patients. They will identify, screen and recruit cancer patients to the study. Communication with the clinical care teams and patient organisations will take place by phone and email. Due to COVID-19 restrictions, potential participants can be contacted if after reading the study advertisement or invitation letter they have e-mailed the lead investigator or have verbally communicated with a member of the clinical care team to explicitly express their interest in the study. Participants will be given a minimum of 24 hours to decide if they wish to take part in the study. No participant will be contacted by the research team without their prior permission.
- After participants have expressed their interest in the study, the lead researcher will provide them with an electronic study information pack (including invitation letter, expression of interest form, information sheet, eligibility criteria and consent form). The researcher will give potential participants an opportunity to ask questions and discuss the study before deciding if they wish to consent to participate. The patient pre-screening process will be conducted either by the lead researcher (via the telephone, WhatsApp or Skype) or in person by a member of the clinical care team when they approach the patients. Interviews will only be arranged once the pre-screening process has been conducted and the participant has had chance to discuss the study and eligibility criteria and give their verbal consent.
- Verbal consent will be obtained and recorded with audio recording equipment prior to data collection. Those who consent to take part in the study will individually attend one 60 minute interview which will be digitally recorded.

#### 1.4.3 Interview questions

Patient interview questions focused will focus on: the experience of being diagnosed during the COVID-19 pandemic; management of transport, appointments and finances; access to healthcare services and information; and support seeking behaviours including access to and use of community support and help from family and friends. Examples of interview questions included:

- *When were you first told that you had cancer and that you would need to have cancer treatment?*
- *I am wondering if accessing appointments or travelling to and from the hospital has been easier or harder than you anticipated*.
- *Can you also tell about anything that may have worried you when accessing healthcare services in relation to your diagnosis and treatment?*
- *Where do you seek information regarding your diagnosis and treatment?*
- *What strategies and resources in your community do you use to manage your diagnosis and treatment?*

Interviews with healthcare professionals explored their work experience and communication with elderly patients in rural settings, barriers and facilitators to delivering timely healthcare and recommendations for service improvement. Some examples of interview questions are:

- *How are you currently able to address your patients’ age-related needs in your practice?*
- *In your experience, do you feel your older patients living in rural settings can easily access healthcare services or attend their appointments?*
- *Thinking about your rural older patients’ care needs and barriers to timely information and healthcare– What would improve their healthcare experience?*
- *I am wondering if in your practice you are able to work with other professionals (including local organisations such as Good Neighbour Schemes) to assist in rural areas*.
- *How are you currently able to identify if any of your older patients in rural areas experience loneliness and social isolation?*

### 1.5 Analytic strategy

The interview data will be analysed using the Framework Method [7]. A jointly developed set of codes will be organised into categories to manage and organise the data. The framework will create a new structure for the data, summarising it in a way that can support answering the research questions [8]. This will be achieved by using a Framework Method matrix of summarised data, which will be reviewed during the analysis. Connections within and between participants and categories will be made in order to generate themes from the data set. This process will be influenced both by the original research objectives and by new concepts generated inductively from the data [8].

## 2. Background and Aims

Elderly cancer patients in rural settings are at high risk of health inequalities due to barriers to accessing timely information and care. Rural populations are ageing “faster” than their urban counterparts [9], thus a higher percentage of elderly people are likely to live in rural areas [10]. Two thirds of newly diagnosed patients with different medical conditions in the United Kingdom (UK) are over the age of 65 [11]. In Lincolnshire, over half (52%) of the county’s older population lives in rural areas [10]. The UK has some of the lowest survival rates in Europe for people aged 65 and over (Munro, 2014), partly due to late diagnosis or under treatment [12]. Patients typically present their symptoms at a later stage which reduces the chances of survival.

Frailty, transportation challenges, lack of disposable income and need of assistance with health literacy [13], incontinence, co-morbidities or co-dependency may prevent this patient group from accessing services and attending appointments relating to their diagnosis, treatment and after-care. In addition, social distancing measures introduced to control the spread of COVID-19 and anxieties about contracting the virus have negatively impacted patients’ health-seeking behaviours [14]. Notably, those who were socially vulnerable before the pandemic now experience more isolation and lack of professional advice on how to mitigate treatment side-effects, or how to cope with the emotional and physical impact of cancer after treatment.

Although contact with health providers by telephone or e-mail is available, often these patients are uncomfortable with this, particularly when they are hearing-impaired or not able to use electronic devices. Inadequate communication between people affected by cancer and health providers is a common barrier to cancer care [15], leading to health disparities, poorer adjustment to illness [16] and lower survival [17].

There are knowledge gaps in service provision within the context of COVID-19, for example understanding the potential impact of shielding upon the health seeking behaviour of rural patients aged 65 and over who have been diagnosed with cancer in the previous 6 months. Understanding the impact of older patients’ social circumstances and their health literacy and access to medical services is a matter of urgency in the UK [17,18]. With such knowledge gaps, it is difficult to assess the specific unmet needs of this group in order to improve their health literacy and communication with health providers, reduce health inequality, and empower them to make informed decisions regarding their health. This study aligns with the top key priorities launched by the National Cancer Research Institute and James Lind Alliance in November 2018, to understand how service delivery can take a more personalised approach.

### 2.1 Aims

We aim (a) to identify and explore the potential impact of COVID-19 and shielding upon the needs, concerns and preferences that newly diagnosed elderly patients have in relation to how, when and what they communicate with their health professionals; (b) to understand the facilitators of and barriers to effective and timely access to information and advice from health professionals; (c) map key health seeking behaviours and decisions this group of patients make in relation to their treatment and care after treatment; and finally (d) to explore patients and health professionals’ views on improving health information literacy and access to timely services.

## 3. Ethical and regulatory considerations

NHS ethics and Health Research Authority study approval have been obtained. The study complies with the Data Protection Act (1998) and the General Data Protection Regulation (2018) with regards to the collection, storage, processing and disclosure of personal information. Data protection NHS and Trust policies, the UK legislation and the principles of Caldicott guardian govern any access to patient data. We will follow Good Clinical Practice guidelines as members of the research team have received training provided by the National Institute for Health Research. A data management plan and a study file have been developed and maintained throughout the study to ensure the secure and ethical handling of research data and to mitigate any risk of loss to confidential information. Electronic data will be stored on secure network drives (e.g. OneDrive) which require system user login to access, or stored within the secure EDGE database, and will only be accessible by the immediate study team and regulatory bodies. Data will not be disclosed to third parties. Our study adheres to equality and diversity principles to reduce barriers to participation in research, especially those from protected groups. A risk register will be maintained throughout the study, ensuring any issues are acted upon and addressed promptly.

### 3.1 Patient and Public Involvement/Engagement

Aligning with the NHS Age Equality Practice Guide and Macmillan ‘Recovery Package’ Programme, this project will draw on specialist knowledge and patient experience. The Lincolnshire Research Patient & Public Forum is part of the project steering group and has ensured that our approach is sufficiently sensitive towards the needs of those diagnosed with cancer. One of the members of the Forum is a member of the project core team. In order to strive for a high standard of ethical research practice, and ensure that the data collection process is sensitive and inclusive, the Lincolnshire Patient and Public Forum, a Macmillan representative and a senior HCP with extensive research experience in cancer research reviewed the research pack (interview questions, consent forms, information and debrief sheets) to ensure external validation and quality check. Consideration was given to the nature of the study, terminology and interview guides. The process was guided by review forms completed by all members involved.

## 4. Dissemination

Our aim is to publish at least two academic papers (e.g. Primary Care, BMC Public Health and Psycho-oncology/Psychology and Health) and to present at conferences organised by the British and International Psychosocial Oncology Society. We will produce research briefings and flyers in relation to these academic outputs for distribution across support groups and will give presentations to patient representative groups in rural areas. Finally, we will feedback the Macmillan Clinical Commissioning Group, to explore new ways of delivering health messages as part of a larger service improvement programme, with the ultimate aim of improving services aimed at tackling the patterning of treatment and health inequalities in older patients diagnosed with cancer in rural settings.
